# Sick building syndrome, multiple chemical sensitivity, and related factors: A cross-sectional analysis from the Japan Environment and Children’s Study

**DOI:** 10.1371/journal.pone.0324562

**Published:** 2025-06-04

**Authors:** Yasuaki Saijo, Eiji Yoshioka, Yukihiro Sato, Hiroyuki Shiotsuki, Kentaro Nakanishi, Yasuhito Kato, Ken Nagaya, Satoru Takahashi, Yoshsiya Ito, Atsuko Ikeda, Hiroyoshi Iwata, Takeshi Yamaguchi, Reiko Kishi, the Japan Environment and Children’s Study (JECS) Group

**Affiliations:** 1 Department of Social Medicine, Asahikawa Medical University, Asahikawa, Hokkaido, Japan; 2 Department of Obstetrics and Gynecology, Asahikawa Medical University, Asahikawa, Hokkaido, Japan; 3 Division of Neonatology, The Center for Maternity and Infant Care, Asahikawa Medical University Hospital, Asahikawa, Hokkaido, Japan; 4 Department of Pediatrics, Asahikawa Medical University, Asahikawa, Hokkaido, Japan; 5 Faculty of Nursing, Japanese Red Cross Hokkaido College of Nursing, Kitami, Hokkaido, Japan; 6 Faculty of Health Sciences, Hokkaido University, Sapporo, Hokkaido, Japan; 7 Center for Environmental and Health Sciences, Hokkaido University, Sapporo, Hokkaido, Japan; Ehime University Graduate School of Medicine, JAPAN

## Abstract

Sick building syndrome (SBS) is caused by having unhealthy indoor environments. Multiple chemical sensitivity (MCS) is a chronic condition that is potentially triggered by low-level chemical exposure. Demographic factors, lifestyle factors, and comorbidities have been reported as potential risk factors of both conditions; however, studies on these factors involving large populations in Japan are limited. The aim of this study was to investigate whether demographics, lifestyle, and comorbidities were associated with MCS and SBS in a large Japanese population, and whether autistic traits (Japanese version of the Autism-Spectrum Quotient Short Form, AQ-10-J), psychological distress (Japanese version of the Kessler 6-Item Psychological Distress Scale, K6), and serum total and allergen-specific immunoglobulin E (IgE) levels were related to the outcomes. The participants included 92,387 pregnant women and 48,451 partners. The outcomes were self-reported physician-diagnosed SBS, MCS, and SBS and/or MCS (combined outcome [CO]). Age-adjusted odds ratios (ORs) of total and antigen-specific IgE levels, demographic factors, and lifestyle factors were determined. The proportions of pregnant women with SBS, MCS, and CO were 307 (0.33%), 128 (0.14%), and 415 (0.45%), respectively, while those of their partners were 85 (0.18%), 30 (0.06%), and 112 (0.23%), respectively. Allergic diseases, psychiatric diseases, migraine, and higher psychological distress were associated with significantly higher ORs for SBS, MCS, and CO. Among pregnant women, autoimmune diseases, cancer, kidney diseases, higher physical activity, autistic traits, and total and specific IgE levels were associated with significantly higher ORs for SBS, MCS, and CO. Clinicians should consider common comorbid disorders when treating patients with SBS and MCS, and their protective and deteriorating lifestyles and demographic factors should be clarified.

## Introduction

Sick building syndrome (SBS) is characterized by non-specific symptoms (e.g., headache, eye, nose, or throat irritations; dry cough; dry or itchy skin; dizziness) developed in specific buildings, and its cause was classified as ambient (environment)-related factors and individual-related factors [1,2]. In Japan, SBS, which occurs in home environments and is caused mainly by exposure to volatile organic compounds, has become a major environmental medical issue since the 1990s and is also called “sick house syndrome” [3,4]. Thus, SHS is a type of SBS developed at home, a term that has been used especially in Japan. Multiple chemical sensitivity (MCS), also known as idiopathic environmental intolerance, is a medical condition of unknown etiology triggered by low-level chemical exposure. They involve a large spectrum of organ systems, and the symptoms are reported to be induced by environmental chemicals at doses far below those usually harmful to most persons [5–7].

Exploring the risk factors for SBS and MCS is a developing field, and various comorbidities related to MCS, such as anxiety, depression, psychotic disorders, migraines, asthma, allergies, atopic dermatitis, autoimmune diseases, neurological diseases, gynecological diseases, and cardiopulmonary diseases, have been reported as potential risks [5,8,9]. The demographics of patients with MCS, such as being female, middle-aged, and of high socioeconomic status, have also been considered potential risks [5,10]. SBS is also related to being female, atopy, anxiety, aggressiveness, low sense of coherence, neuroticism, and working in a poor psychosocial environment [11–13]. A recent review showed that SBS and MCS share common symptoms and possibly have the same mechanisms, such as neurogenic inflammation and neural sensitization [14]. Furthermore, they sometimes overlap [3], with 60% of MCS cases in Japan developing from SHS [15]. However, to the best of our knowledge, no studies have been conducted to simultaneously investigate the comorbidities of SBS (or SHS) and MCS in the general population.

Additionally, the interplay between developmental disorders, SBS, and MCS has attracted attention, and increased incidences of autism and attention deficit hyperactivity disorder (ADHD) in children of parents with MCS have been reported [16,17]. A four-country study (the United States, Australia, Sweden, and the United Kingdom) revealed that individuals with chemical sensitivity were more likely to report autism/autism spectrum disorders (ASDs); however, statistical analysis was not performed [18]. Moreover, no report exists on whether developmental disorders are more common comorbidities in patients with SBS or MCS based on statistical analyses.

Thus, the aim of this epidemiological study was to investigate whether demographics, lifestyles, and comorbidities were associated with SBS or MCS among a relatively young child-bearing Japanese population, and to investigate whether autistic traits (Japanese version of the Autism-Spectrum Quotient short form, AQ-10-J) [19], psychological distress (the Japanese version of the Kessler 6-Item Psychological Distress Scale, K6) [20], and serum total and allergen-specific immunoglobulin E (IgE) levels were associated with SBS or MCS.

## Materials and methods

### Participants

**Fig 1:**
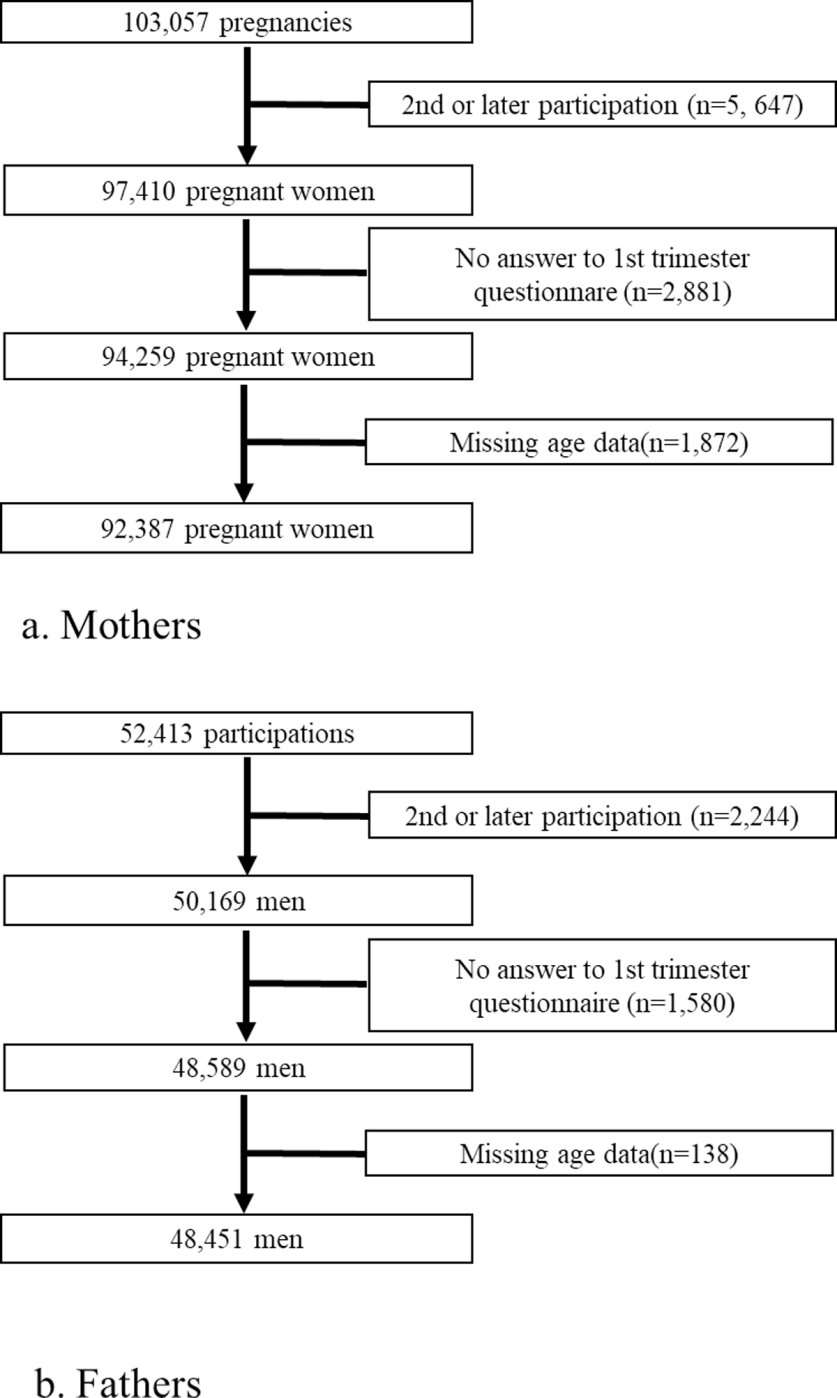
Flowchart of the study.

This study was conducted using data from the Japan Environment and Children’s Study (JECS), an ongoing nationwide, multicenter, prospective birth cohort study. The study was conducted in 15 Regional Centres located in all geographical areas of Japan (Hokkaido, Miyagi, Fukushima, Chiba, Kanagawa, Koshin, Toyama, Aichi, Kyoto, Osaka, Hyogo, Tottori, Kochi, Fukuoka, and South Kyushu/Okinawa) [21,22]. Women at an early stage of pregnancy (103,057 pregnancies) and their partners (52,413) were recruited between January 2011 and March 2014. The total eligible numbers were confirmed, but the child coverage was estimated at approximately 45% in 2013 when recruitment was largely stabilized [22]. Written informed consent was obtained from all participants prior to data collection. After excluding pregnancies involving the same women, 97,410 unique pregnant women were included. After excluding participants with missing first-trimester questionnaire and age data, the final number of pregnant women was 92,387 (Fig 1a). After excluding second participating partners and those with missing the partner questionnaire and age data, 48,451 men were included (Fig 1b). We used the jecs-ta-20190930 dataset from the JECS, which was the JECS registry, the University Hospital Medical Information Network (UMIN) 000030786 (UMIN Clinical Trials Registry).

### Ethics statement

The JECS protocol was reviewed and approved by the Institutional Review Board on Epidemiological Studies of the Ministry of the Environment and the Ethics Committees of all participating institutions. The JECS was conducted by the Declaration of Helsinki and other national regulations. Written informed consent was obtained from all participants.

### History of SBS and MCS

A questionnaire was distributed to the enrolled pregnant women (mothers) during the first trimester (T1 questionnaire for mothers; if participation was delayed, it was distributed during the second or third trimester). Their partners were asked to complete a questionnaire during the mothers’ early pregnancy and one month after delivery. The questionnaire included queries about history of SBS and MCS, and those who answered yes were considered SBS-positive and MCS-positive, respectively.

### Independent variables

The T1 questionnaire for mothers and the partner questionnaire included queries about history of allergies (bronchial asthma, allergic rhinitis or pollinosis, allergic conjunctivitis, atopic dermatitis, food allergy, drug eruption and drug allergy, contact dermatitis), depression, dysautonomia, anxiety disorder, schizophrenia, ADHD, learning disability (LD), ASD (questions about autism, Asperger’s syndrome, and pervasive developmental disorder [PDD]), other development disorders, hypertension, dyslipidemia, cardiovascular diseases, migraine, diabetes, thyroid diseases, other endocrine disorders, autoimmune diseases, digestive diseases, cancer, and kidney diseases. The questionnaire also included questions on smoking (‘never,’ ‘previously did, but quit before realizing current pregnancy,’ or ‘previously did, but quit after realizing current pregnancy/ currently smoking’) and drinking habits (‘nondrinker,’ ‘ex-drinker,’ and ‘drinker’).

T2 questionnaire was distributed to mothers during the second or third trimesters. It comprised questions about maternal and paternal educational attainment, categorized as ≤12 years (≤high school) and ≥13 years (>high school). T2 questionnaire also included questions on annual household income, categorized as ≤3.99 and ≥ 4.00 million yen.

Data on maternal height and pre-pregnancy weight were obtained from medical records transcriptions. If missing, the data were obtained from self-reports. Pre-pregnancy body mass index (BMI) was calculated as maternal pre-pregnancy weight (kg) divided by the square of maternal height (m^2^). The participants were categorized based on their pre-pregnancy BMI as follows: underweight (≤18.5 kg/m^2^), normal weight (18.5–24.9 kg/m^2^), overweight or obese (≥25.0 kg/m^2^). Father’s height and weight were either self-reported or reported by the partner.

Maternal age (≤24, 25–29, 30–34, 35–39, or ≥40 years) and parity (0 or ≥1) were transcribed from medical records. Father’s age was self-reported.

Maternal and paternal psychological distress was assessed using the Japanese version of the K6 in the T1 questionnaire [20,23], with a score ≥5 points indicating positive maternal psychological distress [24]. Maternal and paternal autistic traits were assessed using the AQ-10-J in the T2 questionnaire for mothers or the T1 questionnaire for partners, with a score ≥7 indicating high autism traits.

Maternal physical activity level before pregnancy was evaluated using the Japanese version (short and self-administered) of the International Physical Activity Questionnaire (IPAQ) in the T1 questionnaire, and physical activity level in terms of Met·min/day (metabolic equivalent of a task measured as the number of minute per day) was calculated [25–27]. Physical activity as defined in the IPAQ includes all time spent being physically active, including work-related, housework, and leisure-time activities. Physical activity was quartilized for categorical analysis. IPAQ was not administered to the partners in this cohort.

The mothers’ blood samples were obtained in the first trimester when possible, or in the second trimester if not. Serum total allergen-specific IgE titers were determined in a contract clinical laboratory using immunological assays (ImmunoCAP, Thermo Fisher Scientific, Inc., Sweden). Specific titers were detected for the following allergens: *Dermatophagoides pteronyssinus* (Der p 1), Japanese cedar, egg white, animal dander (from dogs, cats, guinea pigs, rats, and mice), and moth. High serum total IgE levels were defined as ≥170 IU/mL [28,29]. Positivity for allergen-specific IgE sensitization was defined as allergen-specific IgE ≥ 0.35 UA/mL [30–33]. Only total IgE levels were available to the partners in this cohort.

### Statistical analysis

The variables with missing data were shown in Table 1, and 29.94% of mothers and 15.66% of partners had one or more missing values. After stratification by sex (mothers and partners), crude analyses between the abovementioned independent variables and SBS, MCS, and SBS and/or MCS were performed using Fisher’s exact test. Age-adjusted logistic regression models were used to obtain the odds ratios (ORs) of the abovementioned independent variables for SBS, MCS, and SBS and/or MCS (combined outcome, CO). Thus, independent variables were included separately in the age-adjusted logistic regression models, and if the variables had missing values, the total analyzed numbers were reduced.

**Table 1:** Sick building syndrome, multiple chemical sensitivity, and related factors among mothers.

Factors		No SBS/MCS (N = 91,972)		SBS (N = 307)			MCS (N = 128)			SBS and/or MCS (N = 415)		
		N	%	N	%	P	N	%	P	N	%	P
Age (year)	≤24	10,490	11.4	28	9.1	0.078	15	11.7	0.083	41	9.9	0.257
	25-29	26,986	29.3	95	30.9		24	18.8		115	27.7	
	30-34	32,014	34.8	123	40.1		51	39.8		166	40.0	
	35-39	19,176	20.9	56	18.2		32	25.0		82	19.8	
	≥40	3,306	3.6	5	1.6		6	4.7		11	2.7	
Body Mass Index	<18.4	14400	15.7	51	16.6	0.523	26	20.3	0.279	73	17.6	0.272
	18.5-24.9	67600	73.5	218	71.0		87	68.0		291	70.1	
	≥25	9930	10.8	38	12.4		15	11.7		51	12.3	
	Missing	42	0.1	0	0.0		0	0.0		0	0.0	
Parity	0	38162	41.5	143	46.6	0.045	66	51.6	0.023	194	46.8	0.017
	≥1	51526	56.0	152	49.5		59	46.1		206	49.6	
	Missing	2284	2.5	12	3.9		3	2.3		15	3.6	
Education	≤High school	32163	35.0	90	29.3	0.040	39	30.5	0.396	125	30.1	0.042
	>High school	57232	62.2	208	67.8		83	64.8		277	66.8	
	Missing	2577	2.8	9	2.9		6	1.7		13	3.1	
Household income	≤399	33370	36.3	117	38.1	0.542	55	43.0	0.107	166	40.0	0.127
(10,000 yen)	≥400	50072	54.4	163	53.1		61	47.7		212	51.1	
	Missing	8530	9.3	27	8.8		12	9.4		37	8.9	
Smoking	Never	53230	57.9	170	55.4	0.537	61	47.7	0.001	221	53.3	0.014
	Previously did, but quit before realizing current pregnancy	21310	23.2	79	25.7		48	37.5		122	29.4	
	Previously did, but quit after realizing current pregnancy/ currently smoking	16729	18.2	56	18.2		18	14.1		69	16.6	
	Missing	2800	3.0	10	3.3		1	0.8		3	0.7	
Alcohol	Non-drinker	29796	32.4	100	32.6	0.993	43	33.6	0.902	134	32.3	1.000
	Ex-drinker	56923	61.9	189	61.6		75	58.6		255	61.5	
	Drinker	2453	2.7	8	2.6		3	2.3		11	2.7	
	Missing	2800	3.0	10	3.3		7	5.5		15	3.6	
Any allergic diseases	No	45,165	49.1	34	11.1	<0.001	14	10.9	<0.001	47	11.3	<0.001
	Yes	46,807	50.9	273	88.9		114	89.1		368	88.7	
Any allergic diseases (number)*		1	(0-1)	2	(1-3)	<0.001	2	(1-4)	<0.001	2	(1-3)	<0.001
Depression	No	89196	97.0	279	90.9	<0.001	118	92.2	0.006	381	91.8	<0.001
	Yes	2776	3.0	28	9.1		10	7.8		34	8.2	
Dysautonomia	No	88,559	96.3	273	88.9	<0.001	110	85.9	<0.001	366	88.2	<0.001
	Yes	3,413	3.7	34	11.1		18	14.1		49	11.8	
Anxiety disorder	No	89382	97.2	280	91.2	<0.001	113	88.3	<0.001	376	90.6	<0.001
	Yes	2590	2.8	27	8.8		15	11.7		39	9.4	
Schizophrenia	No	91809	99.8	303	98.7	0.002	128	100.0	1.000	411	99.0	0.007
	Yes	163	0.2	4	1.3		0	0.0		4	1.0	
Any psychiatric diseases	No	84793	92.2	243	79.2	<0.001	94	73.4	<0.001	324	78.1	<0.001
	Yes	7179	7.8	64	20.9		34	26.6		91	21.9	
ADHD	No	91944	100.0	307	100.0	1.000	128	100.0	1.000	415	100.0	1.000
	Yes	28	0.0	0	0.0		0	0.0		0	0.0	
Learning disability	No	91958	100.0	307	100.0	1.000	128	100.0	1.000	415	100.0	1.000
	Yes	14	0.0	0	0.0		0	0.0		0	0.0	
ASD	No	91952	100.0	307	100.0	1.000	128	100.0	1.000	415	100.0	1.000
	Yes	20	0.0	0	0.0		0	0.0		0	0.0	
Other development disorders	No	91959	100.0	307	100.0	1.000	128	100.0	1.000	415	100.0	1.000
	Yes	13	0.0	0	0.0		0	0.0		0	0.0	
Any development disorders	No	91904	99.9	307	100.0	1.000	128	100.0	1.000	415	100.0	1.000
	Yes	68	0.1	0	0.0		0	0.0		0	0.0	
Hypertension	No	91542	99.5	306	99.7	1.000	127	99.2	0.452	413	99.5	0.721
	Yes	430	0.5	1	0.3		1	0.8		2	0.5	
Dyslipidemia	No	91527	99.5	305	99.4	0.663	123	96.1	<0.001	408	98.3	0.005
	Yes	445	0.5	2	0.7		5	3.9		7	1.7	
Cardiovascular diseases	No	91808	99.8	307	100.0	1.000	128	100.0	1.000	415	100.0	1.000
	Yes	164	0.2	0	0.0		0	0.0		0	0.0	
Migraine	No	86196	93.7	263	85.7	<0.001	102	79..6	<0.001	351	84.6	<0.001
	Yes	5776	6.3	44	14.3		26	20.3		64	15.4	
Diabetes	No	91782	99.8	306	99.7	0.471	127	99.2	0.233	413	99.5	0.214
	Yes	190	0.2	1	0.3		1	0.8		2	0.5	
Thyroid diseases	No	90774	98.7	302	98.4	0.607	127	99.2	1.000	409	98.6	0.666
	Yes	1198	1.3	5	1.6		1	0.8		6	1.5	
Other endocrine disorders	No	91748	99.8	305	99.4	0.174	127	99.2	0.269	412	99.3	0.083
	Yes	224	0.2	2	0.7		1	0.8		3	0.7	
Autoimmune diseases	No	91363	99.3	302	98.4	0.056	122	95.3	<0.001	404	97.4	<0.001
	Yes	609	0.7	5	1.6		6	4.7		11	2.7	
Digestive diseases	No	78415	85.3	219	71.3	<0.001	87	68.0	<0.001	295	71.1	<0.001
	Yes	13557	14.7	88	28.7		41	32.0		120	28.9	
Cancer	No	90962	97.7	300	98.9	0.088	122	95.3	0.003	403	97.1	0.003
	Yes	1010	2.3	7	1.1		6	4.7		12	2.9	
Kidney diseases	No	90199	98.1	296	96.4	0.056	121	94.5	0.013	398	95.9	0.004
	Yes	1773	1.9	11	3.6		7	5.5		17	4.1	
K6	0-4	61672	67.1	169	55.1	<0.001	59	46.1	<0.001	219	52.8	<0.001
	≥5	29188	31.7	132	43.0		69	53.9		190	45.8	
	Missing	1112	1.2	6	2.0		0	0.0		6	1.5	
K6 (point)*		3	(1-6)	4	(1-7)	<0.001	5	(2-9)	<0.001	4	(2-8)	<.0001
AQ	0-6	85234	92.7	284	92.5	0.855	113	88.3	0.139	379	91.3	0.269
	≥7	2332	2.5	8	2.6		6	4.7		14	3.4	
	Missing	4406	4.8	15	4.9		9	7.0		22	5.3	
AQ (point)*		3	(2-4)	3	(2-4)	0.001	3	(2-4)	0.032	3	(2-4)	<0.001
IPAQ	0- < 28.3	22588	24.6	62	20.2	0.024	17	13.3	0.017	78	18.0	0.004
	28.3- < 115.7	19600	21.3	53	17.3		25	19.5		75	18.1	
	115.7- < 423.4	21312	23.2	71	23.1		36	28.1		101	24.3	
	423.4-17442	21021	22.9	89	29.0		35	27.3		118	28.4	
	Missing	7451	8.1	32	10.4		15	11.7		43	10.4	
IPAQ (point)*		117	(28.3-423.3)	165.4	(37.7-594.0)	0.008	198	(70.7-678)	0.002	171.4	(42.4-605.6)	<0.001
Total IgE	<170	62880	68.4	164	53.4	<0.001	81	63.3	0.049	234	56.4	<0.001
	≥170	19139	20.8	106	34.5		37	28.9		137	33.0	
	Missing	9953	10.8	37	12.1		10	7.8		44	10.6	
Der p1 IgE	<0.35	43557	47.4	56	18.2	<0.001	58	45.3	0.407	110	26.5	<0.001
	≥3.5	38455	41.8	214	69.7		60	46.9		261	62.9	
	Missing	9960	10.8	37	12.1		10	7.8		44	10.6	
Japanese cedar IgE	<0.35	36,545	39.7	77	25.1	<0.001	43	33.6	0.079	116	28.0	<0.001
	≥3.5	45,454	49.4	193	62.9		75	58.6		255	61.5	
	Missing	9,973	10.8	37	12.1		10	7.8		44	10.6	
Egg white IgE	<0.35	81165	88.3	263	85.7	0.023	133	88.3	0.008	360	86.8	0.002
	≥3.5	843	0.9	7	2.3		5	3.9		11	2.7	
	Missing	9964	10.8	37	12.1		710	7.8		44	10.6	
Animal dander IgE	<0.35	65,142	70.8	160	52.1	<0.001	83	64.8	0.022	231	55.7	<0.001
	≥3.5	16,847	18.3	110	35.8		35	27.3		140	33.7	
	Missing	9,983	10.9	37	12.1		10	7.8		44	10.6	
Moth IgE	<0.35	58995	64.1	176	57.3	0.017	80	62.5	0.307	243	58.6	0.006
	≥3.5	22999	25.0	94	30.6		38	29.7		128	30.8	
	Missing	9978	10.9	37	12.1		10	7.8		44	10.6	

* Median (25 percentile - 75 percentile).

SBS: Sick building syndrome (20 MCS overlapping).

MCS: Multiple chemical sensitivity (20 SBS overlapping).

Two-sided P-values <0.05 were considered statistically significant. All analyses were performed using Stata statistical software (version 18.0) for Windows (StataCorp, College Station, TX, USA).

## Results

The proportions of mothers with SBS, MCS, and CO were 307 (0.33%), 128(0.14%), and 415(0.45%), respectively, while those of their partners were 85 (0.18%), 30 (0.06%), and 112 (0.23%), respectively (Tables 1 and 2).

**Table 2:** Sick building syndrome, multiple chemical sensitivity, and related factors among partners.

Factors		No SBS/MCS (N = 48,224)		SBS (N = 85)			MCS (N = 30)			SBS and/or MCS (N = 112)		
		N	%	N	%	P	N	%	P	N	%	P
Age (year)	≤24	3377	7.0	6	7.1	0.696	2	6.7	0.889	8	7.1	0.984
	25-29	11371	23.5	19	22.4		7	23.3		25	22.3	
	30-34	15837	32.8	31	36.5		8	26.7		38	33.9	
	35-39	11851	24.5	19	22.4		8	26.7		26	23.2	
	≥40	5,903	12.2	10	11.8		5	16.7		15	13.4	
Body Mass Index	<18.4	1,652	3.4	1	1.2	0.503	0	0.0	0.275	1	0.9	0.381
	18.5-24.9	32,947	68.2	62	72.9		18	60.0		78	69.6	
	≥25	13,283	27.5	21	24.7		12	40.0		32	28.6	
	Missing	457	1.0	1	1.2		0	0.0		1	0.9	
Education	≤High school	20,032	41.4	23	27.1	0.007	11	36.7	0.584	33	29.5	0.012
	>High school	27,466	56.8	60	70.6		19	63.3		77	68.8	
	Missing	841	1.7	2	2.4		0	0.0		2	1.8	
Household income	≤399	17494	36.2	31	36.5	0.723	13	43.3	0.570	42	37.5	0.611
(10,000 yen)	≥400	27344	56.6	44	51.8		16	53.3		59	52.7	
	Missing	3501	7.2	10	11.8		1	3..33		11	9.8	
Smoking	Never	14068	29.1	33	38.8	0.132	7	23.3	0.681	39	34.8	0.392
	Previously did, but quit before realizing current pregnancy	11521	23.8	21	24.7		6	20.0		27	24.1	
	Previously did, but quit after realizing current pregnancy/ currently smoking	21859	45.2	31	36.5		16	53.3		45	40.2	
	Missing	891	1.8	0	0.0		1	3.3		1	0.9	
Alcohol	Non-drinker	10245	21.2	12	14.1	0.041	4	13.3	0.134	16	14.3	0.009
	Ex-drinker	1806	3.7	7	8.2		3	10.0		10	8.9	
	Drinker	36120	74.7	66	77.7		23	76.7		86	76.8	
	Missing	168	0.4	0	0.0		0	0.0		0	0.0	
Any allergic diseases	No	27,615	57.1	16	18.8	<0.001	10	33.3	0.010	26	23.2	<0.001
	Yes	20,724	42.9	69	81.2		20	66.7		86	76.8	
Any allergic diseases (number)*		0	(0-1)	1	(1-2)	<0.001	1.5	(0-3)	<0.001	1	(1-2)	<0.001
Depression	No	47593	98.5	80	94.1	0.010	27	90.0	0.011	104	92.9	<0.001
	Yes	746	1.5	5	5.9		3	10.0		8	7.1	
Dysautonomia	No	47,853	99.0	81	95.3	0.011	30	100.0	1.000	108	96.4	0.027
	Yes	486	1.0	4	4.7		0	0.0		4	3.6	
Anxiety disorder	No	47868	99.0	82	96.5	0.051	28	93.3	0.035	107	95.5	0.005
	Yes	471	1.0	3	3.5		2	6.7		5	4.5	
Schizophrenia	No	48309	99.9	84	98.8	0.053	30	100.0	1.000	111	99.1	0.069
	Yes	30	0.1	1	1.2		0	0.0		1	0.9	
Any psychiatric diseases	No	46803	96.8	74	87.1	<0.001	26	86.7	0.015	97	86.6	<0.001
	Yes	1536	3.2	11	12.9		4	13.3		15	13.4	
ADHD	No	48323	100.0	85	100.0	1.000	30	100.0	1.000	112	100.0	1.000
	Yes	16	0.0	0	0.0		0	0.0		0	0.0	
Learning disability	No	48335	100.0	85	100.0	1.000	30	100.0	1.000	112	100.0	1.000
	Yes	4	0.0	0	0.0		0	0.0		0	0.0	
ASD	No	48328	100.0	85	100.0	1.000	30	100.0	1.000	112	100.0	1.000
	Yes	11	0.0	0	0.0		0	0.0		0	0.0	
Other development disorders	No	48327	100.0	85	100.0	1.000	30	100.0	1.000	112	100.0	1.000
	Yes	12	0.0	0	0.0		0	0.0		0	0.0	
Any development disorders	No	48300	99.9	85	100.0	1.000	30	100.0	1.000	112	100.0	1.000
	Yes	39	0.1	0	0.0		0	0.0		0	0.0	
Hypertension	No	47118	97.5	83	97.7	1.000	25	83.3	0.001	106	64.6	0.066
	Yes	1221	2.5	2	2.4		5	16.7		6	5.4	
Dyslipidemia	No	47267	97.8	80	94.1	0.041	28	93.3	0.143	105	93.8	0.013
	Yes	1072	2.2	5	5.9		2	6.7		7	6.3	
Cardiovascular diseases	No	48143	99.6	84	98.8	0.293	30	100.0	1.000	111	99.1	0.367
	Yes	196	0.4	1	1.2		0	0.0		1	0.9	
Migraine	No	46696	96.6	69	81.2	<0.001	26	86.7	0.018	93	83.0	<0.001
	Yes	1643	3.4	16	18.8		4	13.3		19	17.0	
Diabetes	No	48032	99.4	85	100.0	1.000	29	96.7	0.174	111	99.1	0.511
	Yes	307	0.6	0	0.0		1	3.3		1	0.9	
Thyroid diseases	No	48176	99.7	85	100.0	1.000	30	100.0	1.000	112	100.0	1.000
	Yes	163	0.3	0	0.0		0	0.0		0	0.0	
Other endocrine disorders	No	48279	99.9	85	100.0	1.000	30	100.0	1.000	112	100.0	1.000
	Yes	60	0.1	0	0.0		0	0.0		0	0.0	
Autoimmune diseases	No	48188	99.7	85	100.0	1.000	30	100.0	1.000	112	100.0	1.000
	Yes	151	0.3	0	0.0		0	0.0		0	0.0	
Digestive diseases	No	40727	84.3	60	70.6	0.001	21	70.0	0.043	79	70.5	<0.001
	Yes	7612	15.8	25	29.4		9	30.0		33	29.5	
Cancer	No	48183	99.7	85	100.0	1.000	30	100.0	1.000	112	100.0	1.000
	Yes	156	0.3	0	0.0		0	0.0		0	0.0	
Kidney diseases	No	47710	98.7	84	98.8	1.000	30	100.0	1.000	111	99.1	1.000
	Yes	629	1.3	1	1.2		0	0.0		1	0.9	
K6	0-4	37554	77.7	55	64.7	0.003	13	43.3	<0.001	67	59.8	<0.001
	≥5	10177	21.1	30	35.3		17	56.7		45	40.2	
	Missing	608	1.3	0	0.0		0	0.0		0	0.0	
K6 (point)*		1	(0-4)	3	(1-7)	<0.001	5	(2-11)	<0.001	3.5	(1-8)	<0.001
AQ	0-6	43774	90.6	74	87.1	0.407	24	80.0	0.043	96	85.7	0.172
	≥7	3173	6.6	7	8.2		5	16.7		11	9.8	
	Missing	1392	2.9	4	4.7		1	3.3		5	4.5	
AQ (point)*		3	(2-5)	4	(3-5)	0.502	4	(3-6)	0.121	4	(2-5)	0.155
Total IgE	<170	31577	65.3	44	51.8	0.017	18	60.0	0.555	61	54.5	0.555
	≥170	15286	31.6	37	43.5		11	36.7		46	41.1	
	Missing	1476	3.1	4	4.7		1	3.3		5	4.5	

* Median (25 percentile - 75 percentile).

SBS: Sick building syndrome (3 MCS overlapping).

MCS: Multiple chemical sensitivity (3 SBS overlapping).

Table 3 shows adjusted ORs for SBS, MCS, and CO among mothers. Parity of one or more was associated with significantly higher ORs for all three outcomes. Compared with ‘never’, ‘previously did, but quit before realizing current pregnancy’ was associated with significantly higher ORs for MCS and CO. Allergic diseases (yes/no, and per number increase), depression, dysautonomia, anxiety disorder, schizophrenia (except for MCS because its OR was not calculable), psychiatric diseases, migraine, autoimmune diseases, digestive diseases, cancer, and kidney diseases were associated with significantly higher ORs for all three outcomes. Dyslipidemia was associated with significantly higher ORs for MCS and CO. K6 score (≥5/ < 5, and per point increase) was associated with significantly higher ORs for all three outcomes. Dichotomous AQ showed no significant results, while continuous AQ (per point increase) was associated with significantly higher ORs for all three outcomes. The highest IPAQ category was associated with significantly higher ORs for all three outcomes, while the second highest IPAQ category was associated with significantly higher ORs for MCS and CO. Per IPAQ score increase was associated with significantly higher OR for all three outcomes. Regarding total and specific IgE levels, almost all allergens were associated with significantly higher ORs for all three outcomes, except for MCS outcome analyses of Der p1, Japanese cedar, and moth IgE levels.

**Table 3:** Age-adjusted odds ratios of sick building syndrome, multiple chemical sensitivity, and related factors among mothers.

Factors		SBS (N = 307)			MCS (N = 128)			SBS and/or MCS (N = 415)		
		OR	95%CI	P	OR	95%CI	P	OR	95%CI	P
Age (year)	≤24	1.00			1.00			1.00		
	25-29	1.32	0.86-2.01	0.199	0.62	0.33-1.19	0.149	1.09	0.76-1.56	0.635
	30-34	1.44	0.95-2.17	0.082	1.11	0.63-1.98	0.713	1.33	0.94-1.87	0.106
	35-39	1.09	0.69-1.72	0.698	1.17	0.63-2.16	0.622	1.09	0.75-1.59	0.639
	≥40	0.57	0.22-1.47	0.242	1.27	0.49-3.27	0.622	0.85	0.44-1.66	0.636
Body Mass Index	<18.4	1.10	0.81-1.49	0.561	1.44	0.93-2.24	0.101	1.18	0.91-1.53	0.199
	18.5-24.9	1.00			1.00			1.00		
	≥25	1.20	0.85-1.69	0.303	1.15	0.66-1.99	0.618	1.20	0.89-1.61	0.241
Parity	≥1	0.77	0.61-0.98	0.032	0.60	0.42-0.86	0.005	0.76	0.62-0.93	0.007
Education	≤High school	1.28	0.99-1.65	0.060	1.15	0.77-1.71	0.488	1.22	0.98-1.51	0.080
Household income	≥400	0.93	0.73-1.19	0.575	0.68	0.47-0.99	0.043	1.22	0.98-1.51	0.080
Smoking	Never	1.00			1.00			1.00		
	Previously did, but quit before realizing current pregnancy	1.16	0.89-1.52	0.273	1.94	1.33-2.83	0.001	1.38	1.10-1.72	0.005
	Previously did, but quit after realizing current pregnancy/ currently smoking	1.07	0.79-1.45	0.682	0.96	0.56-1.63	0.877	1.01	0.77-1.33	0.944
Alcohol	Non-drinker	1.00			1.00			1.00		
	Ex-drinker	0.99	0.78-1.27	0.958	0.92	0.63-1.33	0.648	1.00	0.81-1.23	0.997
	Drinker	0.99	0.48-2.03	0.969	0.81	0.25-2.62	0.728	1.00	0.54-1.85	0.990
Any allergic diseases	Yes (vs. no)	7.69	5.38-10.99	<0.001	7.85	4.5-13.68	<0.001	7.51	5.55-10.18	<0.001
Any allergic diseases (number)*	(per 1 increase)	2.24	2.10-2.40	<0.001	2.48	2.24-2.75	<0.001	2.25	2.12-2.39	<0.001
Depression	Yes (vs. no)	3.21	2.17-4.74	<0.001	2.73	1.43-5.21	0.002	2.86	2.01-4.07	<0.001
Dysautonomia	Yes (vs. no)	3.24	2.26-4.63	<0.001	4.22	2.56-6.95	<0.001	3.47	2.57-4.69	<0.001
Anxiety disorder	Yes (vs. no)	3.33	2.24-4.96	<0.001	4.59	2.67-7.87	<0.001	3.58	2.57-5.00	<0.001
Schizophrenia	Yes (vs. no)	7.28	2.68-19.77	<0.001	*			5.45	2.01-14.76	0.001
Any psychiatric diseases	Yes (vs. no)	3.11	2.36-4.10	<0.001	4.27	2.88-6.32	<0.001	3.31	2.62-4.19	<0.001
ADHD	Yes (vs. no)	*			*			*		
Learning disability	Yes (vs. no)	*			*			*		
ASD	Yes (vs. no)	*			*			*		
Other development disorders	Yes (vs. no)	*			*			*		
Any development disorders	Yes (vs. no)	*			*			*		
Hypertension	Yes (vs. no)	0.75	0.11-5.38	0.777	1.52	0.21-10.93	0.678	1.06	0.26-4.27	0.936
Dyslipidemia	Yes (vs. no)	1.40	0.35-5.65	0.636	7.70	3.12-19.02	<0.001	3.54	1.67-7.54	0.001
Cardiovascular diseases	Yes (vs. no)	*			*			*		
Migraine	Yes (vs. no)	2.50	1.82-3.45	<0.001	3.85	2.50-5.94	<0.001	2.73	2.09-3.57	<0.001
Diabetes	Yes (vs. no)	1.59	0.22-11.41	0.643	3.72	0.52-26.78	0.192	2.34	0.58-9.47	0.232
Thyroid diseases	Yes (vs. no)	1.26	0.52-3.07	0.605	0.56	0.08-4.03	0.567	1.10	0.49-2.47	0.817
Other endocrine disorders	Yes (vs. no)	2.73	0.67-11.04	0.159	3.04	0.42-21.84	0.270	2.96	0.94-9.31	0.063
Autoimmune diseases	Yes (vs. no)	2.54	1.04-6.17	0.040	7.04	3.08-16.07	<0.001	4.10	2.24-7.51	<0.001
Digestive diseases	Yes (vs. no)	2.36	1.84-3.03	<0.001	2.67	1.84-3.88	<0.001	2.37	1.91-2.93	<0.001
Cancer	Yes (vs. no)	2.15	1.01-4.57	0.046	4.21	1.85-9.60	0.001	2.69	1.51-4.8	0.001
Kidney diseases	Yes (vs. no)	1.91	1.04-3.49	0.036	2.86	1.33-6.14	0.007	2.17	1.33-3.54	0.002
K6	≥5	1.66	1.32-2.09	<0.001	2.55	1.80-3.62	<0.001	1.86	1.53-2.26	<0.001
K6 (point)	(per 1 increase)	1.07	1.05-1.10	<0.001	1.12	1.09-1.16	<0.001	1.08	1.06-1.11	<0.001
AQ	≥7	1.03	0.51-2.08	0.935	1.98	0.87-4.51	0.103	1.36	0.80-2.32	0.262
AQ (point)	(per 1 increase)	1.10	1.03-1.18	0.003	1.12	1.01-1.24	0.027	1.11	1.05-1.18	<0.001
IPAQ	0- < 28.3	1.00			1.00			1.00		
	28.3- < 115.7	0.98	0.68-1.41	0.914	1.69	0.91-3.13	0.096	1.10	0.80-1.52	0.545
	115.7- < 423.4	1.21	0.86-1.70	0.281	2.26	1.27-4.02	0.006	1.37	1.02-1.84	0.038
	423.4-17442	1.55	1.12-2.14	0.009	2.30	1.29-4.13	0.005	1.64	1.23-2.19	0.001
IPAQ category	(per 1 category increase)	1.17	1.05-1.30	0.004	1.29	1.09-1.53	0.003	1.19	1.08-1.3	<0.001
Total IgE	≥170	2.12	1.66-2.71	<0.001	1.53	1.03-2.26	0.033	1.93	1.56-2.39	<0.001
Der p1 IgE	≥3.5	4.30	3.20-5.77	<0.001	1.19	0.83-1.70	0.357	2.69	2.15-3.36	<0.001
Japanese cedar IgE	≥3.5	2.01	1.54-2.62	<0.001	1.40	0.96-2.04	0.076	1.76	1.42-2.2	<0.001
Egg white IgE	≥3.5	2.56	1.20-5.43	0.015	4.27	1.74-10.49	0.002	2.94	1.61-5.38	<0.001
Animal dander IgE	≥3.5	2.64	2.07-3.37	<0.001	1.68	1.13-2.49	0.011	2.35	1.90-2.91	<0.001
Moth IgE	≥3.5	1.37	1.06-1.76	0.015	1.23	0.83-1.81	0.301	1.35	1.09-1.68	0.006

* Not calculable.

SBS: Sick building syndrome (20 MCS overlapping).

MCS: Multiple chemical sensitivity (20 SBS overlapping).

Table 4 shows adjusted ORs for SBS, MCS, and CO among partners. Lower educational attainment (≤high school) was associated with significantly higher ORs for SBS. Compared with ‘never’, ‘previously did, but quit after realizing current pregnancy/ currently smoking’ was associated with a significantly lower OR for MCS. Allergic diseases (yes/no, and per number increase), depression, dysautonomia (except for MCS because its OR was not calculable), anxiety disorder, schizophrenia (except for MCS because its OR was not calculable), psychiatric diseases, and migraines were associated with significantly higher ORs for all three outcomes. Digestive diseases and dyslipidemia were associated with significantly higher ORs for SBS and CO. Dichotomous K6 score was associated with significantly higher ORs for all three outcomes, whereas continuous K6 score (per point increase) was associated with significantly higher ORs for SBS and CO. Dichotomous and continuous AQ (per point increase) were associated with significantly higher ORs for MCS. Total IgE levels were associated with significantly higher ORs for SBS and CO.

**Table 4:** Age-adjusted odds ratio of sick building syndrome, multiple chemical sensitivity, and related factors among partners.

Factors		SBS (N = 85)			MCS (N = 30)			SBS and/or MCS (N = 112)		
		OR	95%CI	P	OR	95%CI	P	OR	95%CI	P
Age (year)	≤24	1.00			1.00			1.00		
	25-29	0.94	0.38-2.36	0.896	1.04	0.22-5.01	0.962	0.93	0.42-2.06	0.854
	30-34	1.10	0.46-2.64	0.828	0.85	0.18-4.02	0.841	1.01	0.47-2.17	0.974
	35-39	0.90	0.36-2.26	0.826	1.14	0.24-5.37	0.869	0.93	0.42-2.05	0.850
	≥40	0.95	0.35-2.63	0.927	1.43	0.28-7.38	0.669	1.07	0.45-2.53	0.873
Body Mass Index	<18.4	0.32	0.04-2.33	0.261	*			0.26	0.04-1.85	0.176
	18.5-24.9	1.00			1.00			1.00		
	≥25	0.84	0.51-1.39	0.504	1.62	0.78-3.38	0.196	1.02	0.67-1.54	0.943
Education	≤High school	1.96	1.20-3.19	0.007	1.27	0.60-2.71	0.529	1.75	1.15-2.65	0.009
Household income	≥400	0.90	0.56-1.45	0.677	0.75	0.35-1.59	0.445	0.88	0.59-1.33	0.554
Smoking	Never	1.00			1.00			1.00		
	Previously did, but quit before realizing current pregnancy	0.78	0.45-1.36	0.382	1.01	0.34-3.01	0.989	0.84	0.51-1.38	0.493
	Previously did, but quit after realizing current pregnancy/ currently smoking	0.60	0.37-0.98	0.043	1.50	0.61-3.65	0.374	0.74	0.48-1.14	0.174
Alcohol	Non-drinker	1.00			1.00			1.00		
	Ex-drinker	3.33	1.31-8.48	0.012	4.16	0.93-18.64	0.063	3.53	1.60-7.80	0.002
	Drinker	1.56	0.84-2.88	0.158	1.63	0.56-4.71	0.369	1.52	0.89-2.60	0.123
Any allergic diseases	Yes (vs. no)	5.76	3.34-9.94	<0.001	2.72	1.27-5.83	0.010	4.45	2.87-6.91	<0.001
Any allergic diseases (number)*	(per 1 increase)	2.10	1.80-2.45	<0.001	2.58	2.06-3.25	<0.001	2.13	1.87-2.44	<0.001
Depression	Yes (vs. no)	4.02	1.62-9.96	0.003	6.93	2.09-23.02	0.002	4.90	2.37-10.11	<0.001
Dysautonomia	Yes (vs. no)	4.89	1.78-13.4	0.002	*			3.64	1.33-9.92	0.012
Anxiety disorder	Yes (vs. no)	3.72	1.17-11.81	0.026	7.22	1.71-30.44	0.007	4.74	1.92-11.68	0.001
Schizophrenia	Yes (vs. no)	18.93	2.55-140.56	0.004	*			14.36	1.94-106.3	0.009
Any psychiatric diseases	Yes (vs. no)	4.57	2.42-8.64	<0.001	4.61	1.6-13.28	0.005	4.72	2.73-8.16	<0.001
ADHD	Yes (vs. no)	*			*			*		
Learning disability	Yes (vs. no)	*			*			*		
ASD	Yes (vs. no)	*			*			*		
Other development disorders	Yes (vs. no)	*			*			*		
Any development disorders	Yes (vs. no)	*			*			*		
Hypertension	Yes (vs. no)	0.95	0.23-3.9	0.941	7.56	2.77-20.65	<0.001	2.18	0.94-5.03	0.070
Dyslipidemia	Yes (vs. no)	2.89	1.15-7.27	0.024	2.90	0.67-12.55	0.154	2.96	1.36-6.48	0.006
Cardiovascular diseases	Yes (vs. no)	3.00	0.41-21.81	0.277	*			2.18	0.30-15.74	0.441
Migraine	Yes (vs. no)	6.61	3.82-11.41	<0.001	4.42	1.54-12.69	0.006	5.83	3.55-9.59	<0.001
Diabetes	Yes (vs. no)	*			4.83	0.6436.76	0.128	1.37	0.199.93	0.756
Thyroid diseases	Yes (vs. no)	*			*			*		
Other endocrine disorders	Yes (vs. no)	*			*			*		
Autoimmune diseases	Yes (vs. no)	*			*			*		
Digestive diseases	Yes (vs. no)	2.32	1.44-3.74	0.001	2.23	1.00-4.97	0.050	2.28	1.51-3.46	<0.001
Cancer	Yes (vs. no)	*			*			*		
Kidney diseases	Yes (vs. no)	0.91	0.13-6.59	0.930	*			0.68	0.09-4.88	0.701
K6	≥5	2.01	1.29-3.14	0.002	4.93	2.39-10.16	<0.001	2.49	1.71-3.64	<0.001
K6 (point)	(per 1 increase)	1.07	1.04-1.11	<0.001	1.07	0.99-1.16	0.111	1.06	1.01-1.10	0.012
AQ	≥7	1.30	0.6-2.83	0.503	2.89	1.1-7.58	0.031	1.58	0.85-2.96	0.151
AQ (point)	(per 1 increase)	1.05	0.93-1.18	0.466	1.22	1.01-1.47	0.043	1.08	0.98-1.20	0.133
Total IgE	≥170	1.75	1.13-2.72	0.012	1.28	0.60-2.72	0.516	1.57	1.07-2.31	0.020

* Not calculable.

SBS: Sick building syndrome (3 MCS overlapping).

MCS: Multiple chemical sensitivity (3 SBS overlapping).

## Discussion

This study showed that allergic diseases, psychiatric diseases, migraine, and psychological distress were related to physician-diagnosed SBS, MCS, and CO in men and women, with similar significant relationships among them. Among pregnant women, autoimmune diseases, cancer, kidney diseases, physical activity evaluated using the IPAQ, autistic traits assessed using the AQ-10-J, and total and specific IgE levels were associated with physician-diagnosed SBS, MCS, and CO. To our knowledge, this is the first study to reveal associations of various comorbidities and IgE levels with SBS and MCS in a large Japanese population, although the participants were restricted to pregnant women and their partners.

Patients with MCS may have more comorbidities of allergic diseases [34,35], as one of the proposed mechanisms of MCS development is allergic responses and alterations in the immune system [5]. A relationship between allergies and SBS has been reported [1], and the environmental factors associated with SBS development include mold, fungi, and mites, which can act as allergens [4,36]. In the allergen-specific IgE analysis among mothers, Der p1 IgE had the highest OR for MCS and egg whites had the highest OR for SBS. Because mite allergen is a sick building-related factor, having the highest OR was plausible. A Japanese web survey study revealed that food allergy was significantly related to chemical intolerance, with a higher OR than other allergic diseases [37]. However, the mechanism was unknown, and the mechanism behind egg white IgE having the highest OR for MCS was not plausible in our study. Further studies are required to explore the relationship between MCS and food allergy.

Previous studies have reported associations of comorbidities with SBS [12,38,39] and MCS [5,8,9,40]. In the present study, psychiatric diseases and psychological distress evaluated using the K6 were significantly related to all three outcomes. Although the etiology is unknown, several studies have speculated that MCS and psychiatric diseases have common risk factors [5] and that MCS is possibly caused by mental illness [41,42] and precedes the onset of mental illness [43,44]. Another possible cause of MCS is neurogenic inflammation, which causes mental illness [5,7,8]. Regarding SBS, air pollution has been reported to be associated with poor mental health [7], and one of the risk factors of SBS is psychosocial stress [13], which can cause mental illness.

Migraine is an MCS-related comorbidity [45,46], with common possible mechanisms including central sensitization, oxidative stress, and systemic inflammation [7]. SBS has also been reported to be related to migraines [47]. A reason for these significant relationships may be the overlap between the causes of SBS and migraine triggers such as ambient air pollution and volatile molecules (odorants) [48,49].

Developmental disorders showed no significant results in our study because their prevalence was low, and neither SBS- nor MCS-positive participants had developmental disorders. However, AQ point increase was associated with significantly higher ORs for all three outcomes among mothers, and AQ ‘positive’ and point increase were associated with significantly higher ORs for MCS among partners. Individuals with chemical sensitivity have been reported to be more likely to report autism/ASD [18]. Although our study did not show significant results regarding developmental disorder analyses, significant AQ results may indicate that MCS patients may have an autism spectrum tendency.

Digestive diseases were significantly associated with the outcomes, except MCS, among partners (p = 0.050). In a general Swedish and Finnish adult population study, chemical and building-related intolerance groups had significantly greater digestive symptoms [9]. Gastrointestinal symptoms are common in both MCS and SBS [14]. A review described a hypothesis that MCS development may be attributed to the sensitization of transient receptor potential (TRP) receptors (TRPV1 and TRPA1), and sensitization of TRV1 may be involved in the development of functional dyspepsia and irritable bowel syndromes, both of which are frequently comorbid with MCS [7]. Autoimmune diseases, cancer, and kidney diseases were significantly related to all three outcomes among mothers, and dyslipidemia was significantly related to MCS and CO among mothers and were significantly related to SBS and CO among partners. A previous review suggested that SBS, including MCS, is an autoimmune (autoinflammatory) syndrome induced by adjuvants. However, the mechanisms underlying these associations remain unclear, necessitating further studies.

The prevalence among mothers was higher than that among partners, as previously reported [9,11,39,50]. Regarding smoking status, ‘previously did, but quit before realizing current pregnancy’ was associated with significantly higher ORs for MCS and MCS and/or SBS among mothers, while ‘quitting previously did, but quit after realizing current pregnancy/ currently smoking’ was associated with significantly lower ORs for SBS among partners. Tobacco smoke triggers MCS symptoms, but the smoking status of patients with MCS has been inconsistent across studies [46,51]. Our results may reflect smoke-avoiding behaviors after MCS development among mothers with MCS, and the low OR of quitting during early pregnancy/still smoking among partners may make them less prone to developing environmental exposure-induced symptoms. Patients with MCS have lower levels of alcohol use and higher levels of alcohol intolerance [7]. The higher ORs for ex-drinkers among partners may reflect this tendency. Parity of one or more had a significant protective relationship with the three outcomes. This may be due to healthier mothers having more than one child. MCS has been associated with having a sedentary lifestyle [51], whereas other studies on MCS and SBS have revealed no relationship with exercise [9,39]. In our study, higher physical activity levels were associated with all three outcomes. Further studies are required to clarify whether lifestyle factors are associated with MCS and SBS.

Our study has several limitations. First, because the participants were restricted to pregnant women and their partners, they appeared healthier and younger than the general population. Second, this was a cross-sectional study; therefore, causal relationships could not be established. As previously mentioned, as psychosocial stress is a common risk factor of both SBS and mental illness [13], common risk factors may have the role for confounders. Furthermore, reverse causations should be considered that suffering diseases like MCS and SHS would cause mental illnesses. Third, MCS and SBS were physician-diagnosed and self-reported. Moreover, there are no specific medical tests to diagnose MCS and SBS; therefore, misclassification may have occurred. Furthermore, SHS and MCS are assumed to be the same among the general Japanese population [52], and they sometimes overlap [3]. A review revealed that the considerable similarities between SBS and MCS suggest the two conditions largely share the same mechanisms [14]. Therefore, “MCS and/or SHS” was defined as a significant outcome in our study. Fourth, because this study was not able to prove the etiology of SBS and MCS, and this was research to explore the various relational factors for SBS and MCS, statistical corrections for multiple comparisons were not done, and only age-adjusted OR were shown after sex-stratification. To confirm the current results, the associations must be tested in further studies. Finally, because this study data were based on a birth cohort study focusing on pregnant women, there were more women than men, and some data, such as antigen-specific IgE levels and IPAQ scores, were not available for the partners.

## Conclusions

Allergic diseases, psychiatric diseases, migraine, and psychological distress were associated with SBS, MCS, and CO among both women and men, and autoimmune diseases, cancer, and kidney diseases were associated with SBS, MCS, and CO among women. Allergy and total and antigen-specific IgE levels were also correlated. Furthermore, AQ was related to the three outcomes among mothers, and MCS among partners. These results indicate possible relationships among autism traits, MCS, and SHS. Clinicians should consider common comorbid disorders when managing patients with SBS and MCS. Mothers had a higher prevalence, and higher physical activity level was related to mothers’ SBS, MCS, and CO, and parity of one or more had a protective relationship. Therefore, it is necessary to identify between protective and deteriorating lifestyles and demographic factors.
